# GOST: A generic ordinal sequential trial design for a treatment trial in an emerging pandemic

**DOI:** 10.1371/journal.pntd.0005439

**Published:** 2017-03-09

**Authors:** John Whitehead, Peter Horby

**Affiliations:** 1 Department of Mathematics and Statistics, Lancaster University, Lancaster, United Kingdom; 2 Centre for Tropical Medicine and Global Health, Nuffield Department of Medicine, University of Oxford, Oxford, United Kingdom; George Washington University School of Medicine and Health Sciences, UNITED STATES

## Abstract

**Background:**

Conducting clinical trials to assess experimental treatments for potentially pandemic infectious diseases is challenging. Since many outbreaks of infectious diseases last only six to eight weeks, there is a need for trial designs that can be implemented rapidly in the face of uncertainty. Outbreaks are sudden and unpredictable and so it is essential that as much planning as possible takes place in advance. Statistical aspects of such trial designs should be evaluated and discussed in readiness for implementation.

**Methodology/Principal findings:**

This paper proposes a generic ordinal sequential trial design (GOST) for a randomised clinical trial comparing an experimental treatment for an emerging infectious disease with standard care. The design is intended as an off-the-shelf, ready-to-use robust and flexible option. The primary endpoint is a categorisation of patient outcome according to an ordinal scale. A sequential approach is adopted, stopping as soon as it is clear that the experimental treatment has an advantage or that sufficient advantage is unlikely to be detected. The properties of the design are evaluated using large-sample theory and verified for moderate sized samples using simulation. The trial is powered to detect a generic clinically relevant difference: namely an odds ratio of 2 for better rather than worse outcomes. Total sample sizes (across both treatments) of between 150 and 300 patients prove to be adequate in many cases, but the precise value depends on both the magnitude of the treatment advantage and the nature of the ordinal scale. An advantage of the approach is that any erroneous assumptions made at the design stage about the proportion of patients falling into each outcome category have little effect on the error probabilities of the study, although they can lead to inaccurate forecasts of sample size.

**Conclusions/Significance:**

It is important and feasible to pre-determine many of the statistical aspects of an efficient trial design in advance of a disease outbreak. The design can then be tailored to the specific disease under study once its nature is better understood.

## Author summary

Since many outbreaks of infectious diseases last only six to eight weeks, there is a need for trial designs that can be implemented rapidly in the face of uncertainty. The Generic Ordinal Sequential Trial (GOST) is a flexible statistical design for a randomised clinical trial comparing an experimental treatment for an emerging infectious disease with standard care. The details of the design are derived to satisfy a generic power requirement using large sample theory. The accuracy of the approach for moderate sample sizes is then checked using million-fold simulations, and found to be very reliable under a wide range of circumstances. Total sample sizes (across both treatments) of between 150 and 300 patients prove to be adequate in many cases, although more patients may be needed if the majority of patients die or if the majority experience complete recovery, as there is then less evidence available to distinguish between treatments. An advantage of the approach is that any erroneous assumptions made at the design stage about the proportion of patients falling into each outcome category have little effect on the error probabilities of the study, although they can lead to inaccurate forecasts of sample size.

## Introduction

The 2013–15 Ebola virus disease epidemic in West Africa highlighted the need to be able to develop treatment trial protocols in a matter of weeks, rather than the months or even years that are more usually taken. Clinical research on epidemic infectious diseases has to take place when new cases are occurring. Urgency arises because the outbreak might subside before any lessons about treatment can be learnt, or worse, the outbreak might spiral out of control before effective therapies can be developed.

This paper presents statistical aspects of trial designs that can be developed in advance and then quickly be adapted for a particular outbreak. The Generic Ordinal Sequential Trial (GOST) is a flexible, off-the-shelf statistical design for a randomised clinical trial comparing an experimental treatment with standard care for an emerging infectious disease. Key aspects of GOST are fixed in advance, so that clinicians and statisticians can immediately adopt these generic features, and focus on the optional elements that have to be determined as well as the countless other tasks involved in initiating a clinical trial of this nature. The context envisaged is one where there are only weeks available for preparation, perhaps with limited knowledge of the natural history of the disease. This paper may also be a helpful illustration for research teams with longer to prepare for a trial. In that case, trial statisticians might wish to vary the fixed elements of the design and to explore the consequences using methods described in [1], perhaps applying the statistical code provided in [2].

The full name of GOST, Generic Ordinal Sequential Trial, includes the statistical terms *ordinal* and *sequential*. An *ordinal* scale is a categorisation of outcomes for which there is an intrinsic ranking (or order) of the categories in terms of desirability, but there is no specific numerical value attached to each one. A clinical trial is *sequential* if it is conducted using a sequence of successive analyses, each of which may resolve the primary clinical question and lead to the termination of the trial. The primary trial endpoint in GOST is an ordinal categorisation of patient outcome as recorded a specified number of days following randomisation, and the sequential monitoring will lead to stopping as soon as it is clear that the experimental treatment has an advantage or that sufficient advantage is unlikely to be detected. The trial is powered to detect a generic clinically relevant difference: namely an odds ratio of 2 for better rather than worse outcomes. Total sample sizes (across both treatments) of between 150 and 300 patients prove to be adequate in many cases, the precise value depending on both the magnitude of the treatment advantage and the nature of the ordinal scale.

## Methods

As many features of GOST as possible are pre-specified so that much of the statistical section of the trial protocol can be developed in advance, before the nature of the disease is known and without details of the experimental treatment. Other elements, such as details of the ordinal outcome scale, the randomisation ratio and the day on which a patient’s primary assessment will be made, will have to be quickly determined by investigators once an outbreak occurs.

Patients will be randomised between an experimental treatment (E) and standard care (S), stratified by treatment centre and perhaps by one or two other key prognostic factors. Usually the allocation ratio will be set to 1:1 for simplicity and because expected sample sizes are minimised if this choice is made [3]. If, however, the availability of E were limited, then the allocation ratio could be modified to randomise more patients to S than to E.

The primary patient response will be the status of the patient, D days after randomization, classified into one of k outcome groups, C_1_, …, C_k_. D is likely to be set at 7, 14 or 28 days. The outcome categories must be unambiguously defined and each patient must fall into exactly one of them. They must also reflect progressively less desirable states as one moves from C_1_ (the best outcome) to C_k_ (the worst outcome). Outcome C_1_ might reflect complete recovery and C_k_ death before Day D. Intermediate outcomes might include C_2_: alive and requiring only basic support and C_3_: alive but requiring intensive support, where these terms would need careful definition for specific diseases. It is not necessary for the number of patients in every outcome category to be large, and the method remains valid and accurate if one or more categories turn out to be completely empty, provided that at least two categories are well represented. The special case k = 2 allows for a binary outcome such as alive or dead.

Use of a response that is available after a short and fixed duration of follow-up reduces the risk of loss to follow-up and is essential if the trial is to yield an early conclusion. GOST is presented for the case of an ordinal response because expected sample sizes will be reduced if more than two outcome categories can be reliably identified [3]. Furthermore, at the outset of a trial concerning a new infection, it may not be clear whether the key issue will be the prevention of death or the reduction of morbidity. Using a categorisation that distinguishes between a number of outcome states will allow the trial to be informative if life or death proves to be the major issue or if fatalities prove to be rare and the need for intensive therapy becomes the key concern. In normal circumstances, a pilot study of conventionally treated patients might be used to determine a binary endpoint for the trial: here we are concerned to start the definitive randomised study as early in the outbreak as possible.

The probability that a patient on E achieves an outcome category that is any one of C_1_,…, C_j_, is denoted by P_Ej_. Achieving an outcome in any one of categories C_1_,…, C_j_ is preferable to being in one of the categories C_j+1_,…, C_k_, an event that occurs with probability 1 –P_Ej_. The odds of the former event is O_Ej_ = P_Ej_/(1 –P_Ej_), and P_Sj_ and O_Sj_ are defined similarly for patients receiving S. The odds ratio R_j_ is defined by R_j_ = O_Ej_/O_Sj_. Notice that these definitions make sense for values of j from 1 to k– 1, but they are not used for j = k as P_Ek_ and P_Sk_ refer to the probability of a patient being in any of the outcome categories, which must be 1, and the corresponding odds values are undefined. The null hypothesis is that E has no effect, in which case P_Ej_ = P_Sj_ and so R_j_ = 1 for each value of j from 1 to k– 1. If this null hypothesis is true then the probability of concluding that E is better than S (an event that will be designated “E wins” hereafter) is set to equal 0.025. This is the one-sided risk of type I error (denoted by α), and the value of 0.025 is chosen for GOST to follow convention.

As well as considering the properties of the design when the treatment has no effect (the null hypothesis), we consider its properties when there is a tendency for patients to achieve better outcome categories on E than on S across the whole outcome scale (the alternative hypothesis). Thus, treatment with E might lead to a greater chance of complete recovery, a greater chance of complete or partial recovery, and a smaller chance of death. Specifically, situations are considered in which all of the odds ratios from R_1_ to R_k−1_ are of equal magnitude (denoted by a common value R) and greater than 1. The design is constructed to ensure that, if R = 2, then the probability that E wins is 0.90. This is the power of the trial. The alternative hypothesis is a compromise between the desires to detect small but worthwhile treatment effects and to complete the trial quickly. For a binary outcome, an odds ratio of 2 corresponds to an increase in success rate from ⅓ on S to ½ on E, or from ½ to ⅔, or from ⅔ to ⅘. Typical sample sizes when GOST is employed are in the range 150–300 (totalled over both treatment groups). The value of 0.90 is chosen for the power of GOST as it is a conventional choice: choosing 0.80 would allow too large a risk of missing a treatment effect as large as R = 2. For an increase in success rate from ½ on S to ⅗ on E (R = 1.5), GOST will conclude that E is better than S with probability 0.47. The sample size would triple to 450–900 if a power of 0.90 were specified for the alternative R = 1.5.

The trial will be monitored using a series of up to 20 interim analyses, equally spaced by newly accrued patient responses. It will be seen in the results section that such a choice will lead to around 20 to 30 new responses (totalled over S and E) being needed between consecutive interim analyses. It will also be seen that typically only 8 to 12 of these analyses will be required before the trial stops. The data requirements for each patient at each interim analysis are modest: patient identification number, date of randomisation, treatment, treatment centre and any other baseline stratification factors, and status on Day D. Setting 20 interim analyses for GOST is a subjective choice of the authors achieving a much quicker reaction to the message of the data than setting just 3 or 4 interim analyses while being more practical than updating the sequential plot every time a Day D report is received.

**Fig 1:**
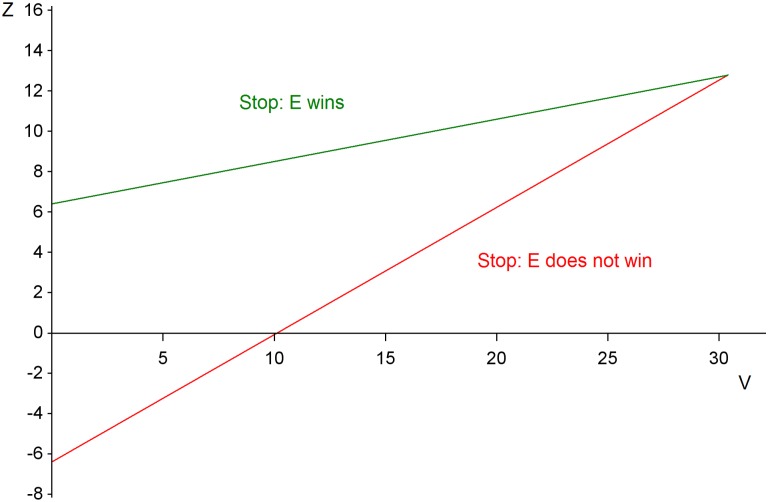
Stopping boundaries for the plot of Z against V. After the i^th^ interim analysis the values Z_i_ and V_i_ are calculated, and Z_i_ is plotted against V_i_ on this figure, i = 1, 2,….

At each interim analysis two test statistics are calculated. The first is a cumulative measure of the observed advantage of E over S and is denoted by Z. The second quantifies the amount of information about the treatment difference contained in Z, and is denoted by V. Expressions for computing Z and V, allowing for stratification factors, are taken from [4] and presented in equations (E1) and (E2) of the Supporting Information (S1 Text. Supporting technical details). The monitoring of GOST can be depicted by a plot of the values of Z computed at each interim analysis against the corresponding values of V, using the diagram shown in Fig 1. A completed plot is presented in the results section. The stopping rule is represented by two straight lines. If Z lies above the upper line, the trial is stopped and E wins. If the plotted value of Z lies below the lower line, the trial is stopped and it is concluded that no evidence that E is better than S has been found. This design is a special case of the triangular test [1, 2], and it was proposed as the phase III part of a trial strategy for Ebola virus disease [5, 6].

**Fig 2:**
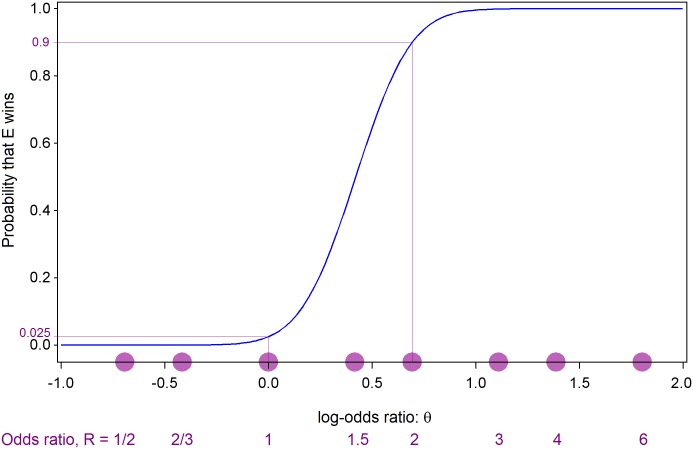
Probability of concluding that the experimental treatment is efficacious (E wins) plotted against the true value of the log-odds ratio θ = ln(R).

**Fig 3:**
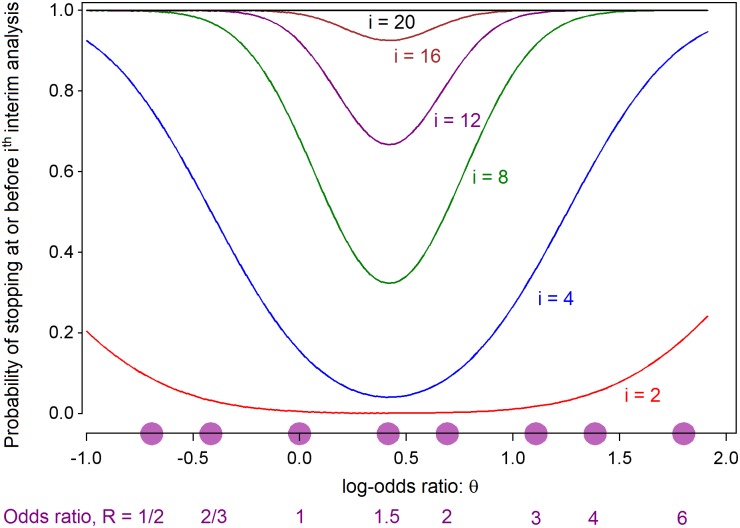
Probability of stopping at or before the i^th^ interim analysis, i = 2, 4, 8, 12, 16, 20; plotted against the true value of the log-odds ratio θ = ln(R).

**Fig 4:**
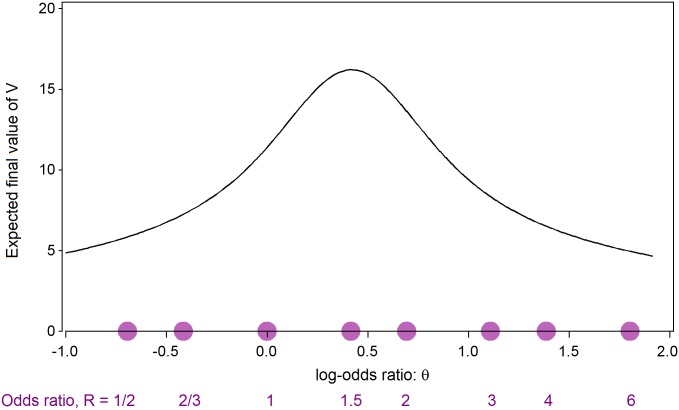
Expected value of the final value of the statistic V plotted against the true value of the log-odds ratio θ = log_e_R.

Fig 2 shows the probability that E wins, plotted against the natural logarithm, θ, of the true odds ratio R. When R = 1 (θ = 0) the plotted probability is 0.025 and when R = 2 (θ = 0.693) it is 0.90. Fig 3 shows the probability of stopping at or before selected interim analyses, plotted against the true value of the log-odds ratio θ. In both of these figures values of θ corresponding to selected values of the odds-ratio R are also indicated on the horizontal axis. Although a maximum of 20 analyses is allowed, it is very unlikely that more than 16 will be required. If the treatment is either harmful (R < 1, θ < 0) or very efficacious (R > 2.7, θ > 1), then it is unlikely that more than 4 interim analyses (one fifth of the maximum sample size) will be needed. Fig 4 shows the expected value of V at the end of the trial (that is the average value of the final value of V over many iterations of the same trial) plotted against θ. As discussed later, the values of V in Fig 4 can be converted into expected final sample sizes. During the trial, V will be calculated according to equation E2 in S1 Text, but prior to the trial starting, the relationship between sample size and V can be approximated using equation E3 in S1 Text to yield a plot of expected terminal sample size against θ, as will be illustrated in the results section below.

The primary analysis will be based on the sequential design used, and will feature a one-sided p-value for the null hypothesis of no treatment difference, and a median unbiased estimate and 95% confidence interval for R. In the final dataset, the numbers of new patients recruited to each treatment arm may not be as planned in the protocol, the allocation ratio might not be as intended and the information V accrued might not be as anticipated. Provided that departures from the plan are purely chance deviations rather than being prompted by emerging data, actual values of these quantities will be used in the analysis. Thus it is acceptable if an unexpected surge of recruitment leads to there being more information available for an interim analysis than anticipated, but it is not acceptable for investigators to see a value of Z close to the stopping boundary and to bring forward the next interim analysis in the hope of a quick conclusion. The valid analysis is described in [1] and statistical code for its implementation is provided in [2]. Conduct of the final analysis will need expert statistical input. Unlike the finalisation of the design, there should be sufficient time for the trial statistician to study and practice these methods ahead of the trial reaching a conclusion. Although the final analysis will require technical input, the conclusion of the trial—whether E wins or not—will be immediately apparent from a glance at the plot of Z against V.

When the trial is stopped, there may still be patients under treatment whose outcome is unknown, as well as patients whose status became available during the conduct of the interim analysis and its discussion. Data from these patients will be added into a final “overrunning” analysis [7], provided that they followed the protocol without any change of treatment due to the stopping of the trial. The latter might not be the case if the experimental treatment is suspected of being harmful and it is consequently withdrawn from current patients.

## Results

Consider comparing an experimental treatment (E) with standard therapy (S) for Middle East Respiratory Syndrome Coronavirus (MERS-CoV) motivated by a sudden increase in the number and geographical spread of incident cases. Randomisation is 1:1. We choose D = 28 days and outcome categories C_1_: alive and not receiving ventilation; C_2_: alive and receiving only non-invasive ventilation; C_3_: alive and receiving invasive mechanical ventilation and C_4_: dead. Data from an observational study [8] of 70 patients yield estimates of the probabilities of these four outcomes occurring for patients on S of 0.286, 0.043, 0.214 and 0.457 respectively.

In Table 1, these four outcome probabilities form Column 2. In the first of 12 sets of simulations, one million replicate runs of GOST were conducted in which these outcome probabilities governed the responses both for patients receiving S and for those receiving E. The results are shown in the second column of Table 2. The proportion of trials in which E won was 0.025; equal to the intended one-sided type I error rate, confirming the accuracy of the procedure. In the second set of simulations, outcome probabilities for patients receiving S were unchanged, but a common odds ratio of R = 1.5 was imposed and the respective probabilities 0.375, 0.048, 0.217 and 0.359 (shown in Column 3 of Table 1, and reflecting a shift to better outcomes) were used to generate patient outcomes on E. For the third set of simulations, the outcome distribution on S was again unchanged, but R was increased to 2. The results are shown in Column 4 of Table 2, showing that the intended power of 0.90 was achieved.

**Table 1:** Scenarios for the evaluation of the trial design.

Probability that a patient on E has the indicated outcome	Scenario 1			Scenario 2			Scenario 3			Scenario 4		
	Odds ratio R:			Odds ratio R:			Odds ratio R:			Odds ratio R:		
	1	1.5	2	1	1.5	2	1	1.5	2	1	1.5	2
C_1_: alive and not receiving ventilation	**0.286**	0.375	0.445	**0.300**	0.391	0.462	**0.550**	0.647	0.710	**0.700**	0.778	0.824
C_2_: alive and receiving non-invasive ventilation	**0.043**	0.048	0.050	**0.050**	0.056	0.057	**0.000**	0.000	0.000	**0.100**	0.079	0.065
C_3_: alive and receiving invasive mechanical ventilation	**0.214**	0.217	0.209	**0.200**	0.200	0.191	**0.000**	0.000	0.000	**0.100**	0.074	0.058
C_4_: dead	**0.457**	0.359	0.296	**0.450**	0.353	0.290	**0.450**	0.353	0.290	**0.100**	0.069	0.053

**Table 2:** Results of million-fold simulations.

	Scenario 1			Scenario 2			Scenario 3			Scenario 4			*Pre-trial forecasts*		
	Odds ratio R:			Odds ratio R:			Odds ratio R:			Odds ratio R:			Odds ratio R:		
	1	1.5	2	1	1.5	2	1	1.5	2	1	1.5	2	1	1.5	2
Proportion of trials where E wins	**0.025**	0.471	0.900	**0.025**	0.472	0.900	**0.026**	0.473	0.898	**0.026**	0.473	0.895	***0*.*025***	*0*.*471*	*0*.*900*
Average final V	**11.45**	16.35	13.82	**11.45**	16.35	13.81	**11.27**	16.04	13.55	**11.20**	15.86	13.46	***11*.*40***	*16*.*21*	*13*.*65*
Average final sample size	**158**	222	187	**158**	222	188	**183**	268	233	**206**	321	291	***157***	*220*	*184*

Nine more simulation runs were conducted. The outcome distributions for patients on S were changed to those shown in bold in Table 1 under Scenario 2, and then as shown for Scenarios 3 and 4. For each scenario, three outcome distributions on E were explored, corresponding to R = 1 (no treatment effect), 1.5 and 2. Scenario 2 uses a rounded version of the estimated distribution on S to demonstrate that precise values are unnecessary at the design stage. Scenario 3 represents a more extreme situation in which all patients either leave intensive care or die by Day 28, while in Scenario 4, most patients leave intensive care by Day 28, with the other three categories being unusual. Values reported in Table 2 for Scenarios 1 and 2 are virtually indistinguishable, but more patients are needed in the case of Scenario 3 or 4.

**Fig 5:**
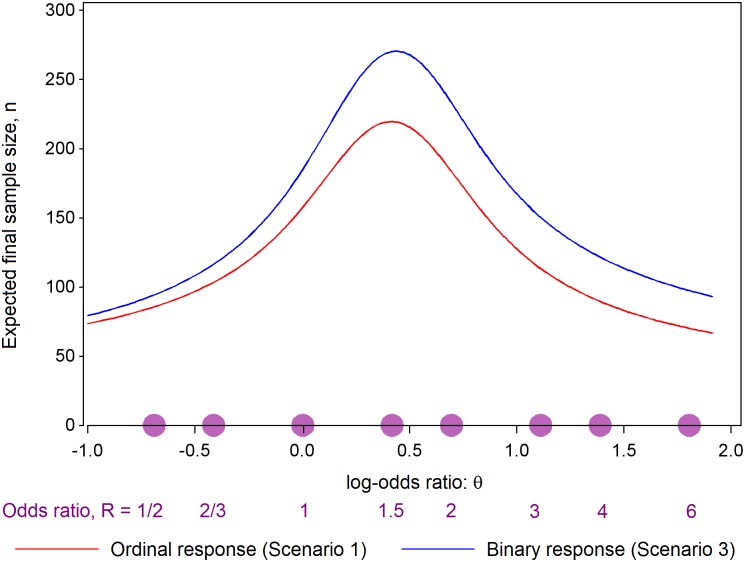
Expected value of the final sample size plotted against the true value of log-odds ratio R, when R_1_ = R_2_ = R_3_ = R, when ordinal responses are to be collected and when binary responses are to be collected.

When interpreting the simulation results shown in Table 2, it is important to distinguish between what the trial designer anticipated as the truth before starting the trial and what was actually true. All simulations represent trials in which the investigators anticipated that Scenario 1 was true, even if they were wrong. As explained in the Supplementary Information (S1 Text), if Scenario 1 is true, then a maximum sample size of 440 will be sufficient to ensure that V eventually reaches the value where the stopping boundaries in Fig 1 meet, so that a conclusion must be reached. Thus, 22 new patient responses will be needed for each of the 20 interim analyses. The expected final values of the information statistic V shown in Fig 4 can be converted into expected final sample sizes under Scenario 1, and the latter are shown as the red curve in Fig 5. The expected sample size lies well below the maximum sample size of 440 whatever the true treatment effect. Investigators’ pre-trial forecasts of the simulated quantities are shown in the last three columns of Table 2.

Having set the design and forecast its properties assuming Scenario 1, the simulations are then conducted under the twelve different models displayed in Table 1. For Scenario 1, with R = 1, 1.5 or 2, the investigators’ predictions are confirmed as being very accurate: average sample sizes are at most 3 over forecast. Switching to Scenario 2 shows how minor imperfections in the anticipated model have negligible effects. Scenarios 3 and 4 are quite different from the design assumptions, and yet the simulated probabilities that E wins and the simulated average final values of V remain close to predictions. The average sample sizes needed to reach a conclusion are, however, considerably larger than anticipated. Being wrong about the underlying model at the design stage will have little effect on the error probabilities of the study, but it might lead to inaccurate forecasts of sample size. The design reacts to the true nature of the data collected to ensure that the appropriate sample size is collected. Note that neither the predictions nor the simulations of average sample sizes include patients who are receiving treatment at the time of analysis, but who have not yet provided a Day 28 response, nor those recruited during the conduct of what turns out to be the final interim analysis.

**Fig 6:**
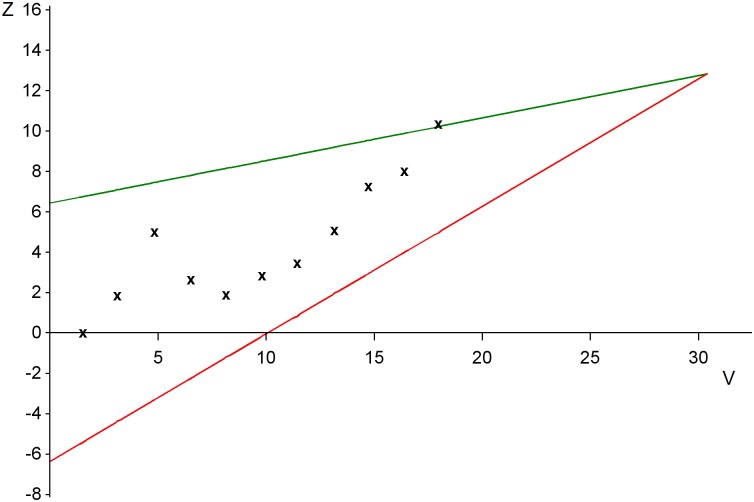
Illustrative plot of Z against V, with stopping boundaries. The trial stops with the conclusion that the experimental treatment is efficacious at the 11^th^ interim analysis.

Table 3 presents data from a single simulated run of GOST and Fig 6 shows the resulting plot. This fictitious trial stopped at the 11^th^ interim analysis with 242 patients, and E won. Using the approach described in [1], the one-sided p-value is found to be 0.016. The median unbiased estimate of the log-odds ratio θ is 0.568 with 95% confidence interval (0.059, 1.062). For the odds-ratio R, the median unbiased estimate is 1.76 with 95% confidence interval (1.06, 2.89). The simulation did not generate patient data that would be received by the investigators after this analysis, but in practice results would come in from study patients who were still being followed to 28 days at the time the data for the 11^th^ interim analysis were extracted, and those who were recruited while that analysis was being undertaken. Provided that no change was made to the treatment of these patients, they could be included in a subsequent overrunning analysis [7], and this would become the definitive interpretation of the trial results.

**Table 3:** Data for the numerical example.

		Standard (S)				Experimental (E)					
i	n_••_	n_S1_	n_S2_	n_S3_	n_S4_	n_E1_	n_E2_	n_E3_	n_E4_	Z_i_	V_i_
1	22	6	0	1	4	5	1	2	3	−0.046	1.540
2	44	9	0	3	10	11	1	4	6	1.796	3.131
3	66	10	0	8	15	17	2	7	7	4.939	4.855
4	88	13	2	11	18	18	2	9	15	2.580	6.539
5	110	18	2	12	23	20	4	11	20	1.827	8.156
6	132	18	5	15	28	24	4	13	25	2.780	9.825
7	154	22	5	17	33	28	4	17	28	3.390	11.456
8	176	24	5	21	38	31	6	21	30	5.017	13.170
9	198	27	5	23	44	38	6	22	33	7.197	14.749
10	220	32	7	23	48	44	7	24	35	7.959	16.410
11	242	32	8	24	57	47	9	25	40	10.285	17.992

The i^th^ row represents the data available at the i^th^ interim analysis, with n_••_ denoting the total number of patient records, n_Sj_ the number of patients on S in Category C_j_, n_Ej_ the number of patients on E in Category C_j_, and Z_i_ and V_i_ denoting the values of the test statistics. (There is only a single stratum, so patient counts n have only 2 subscripts in this example.)

We conclude this section with a brief account of the changes that would follow if the investigators chose to dichotomise patient responses into alive at 28 days (C_1_, C_2_ or C_3_), or dead (C_4_). Taking the rounded outcome probabilities of Scenario 2, and then combining those relating to the first three categories, leads to Scenario 3. GOST can be applied to such binary data, and equations E4 in S1 Text provide simplified versions of the test statistics. However, binary data are less informative than the ordinal version of the data, and it will now take 520 patient responses to ensure that V eventually reaches the value where the stopping boundaries in Fig 1 meet. Thus 26 new responses will be required at each interim analysis. The blue curve of Fig 5 indicates expected final sample sizes for the binary approach, and it can be compared with the red curve that corresponds to both Scenario 1 and Scenario 2, as the two are indistinguishable. The inflation in sample size due to dichotomising the ordinal scale is a factor of 1.18: an 18% increase in sample size. Additional simulations conducted using 26 new binary responses per interim analysis confirmed that the intended type I error rate 0.025 and the power of 0.90 were achieved, but the increase in average final sample sizes relative to those for the ordinal approach reported for Scenarios 1 and 2 in Table 2 ranged from 17% to 26%.

## Discussion

GOST has been devised for trialists in a hurry due to the speed with which a pandemic is emerging. It is intended that they use the GOST design as described in this paper. The investigators have to identify the outcome categories and the day D of their observation. They also choose the allocation ratio and any stratification factors. The rest is as presented above.

Usually, evidence from two or more trials is required for drug registration, although in certain circumstances evidence from just one is considered to be sufficient [9, 10]. It would be important to determine in advance whether a single trial would be sufficient in future outbreaks of infectious diseases. GOST provides an approach that could be used once or repeated in a replicate trial if deemed necessary.

A “platform approach” was suggested for trials of a series of experimental treatments in Ebola virus disease [11]. First a comparison of Treatment E_1_ with S is conducted. If Treatment E_1_ wins it becomes the new standard. Treatment E_2_ is then compared with the current standard, and so on. The α level required to declare a treatment superior to control is fixed at that relevant to a single trial, with no allowance for multiplicity of experimental treatments. GOST could be used as the design for each comparison made within the platform approach, with α being set at 0.025 throughout. Implementations of GOST that allow simultaneous randomisation between multiple experimental treatments and S are also possible.

The triangular test is just one of many sequential methods that could be used as the engine to drive GOST. Alternatives based on α = 0.025 and power 0.90 to detect an odds-ratio of 2 would be natural competitors. The triangular test is chosen because amongst tests satisfying the power requirement above, it minimises the maximum expected sample size, which occurs when R is close to 1.5 [12]. The efficiency of the triangular test is achieved from its asymmetry. Strong evidence is required for E to win, but if superiority is not apparent the trial will stop quickly without recommending E. The design does not seek to distinguish between lack of effect and harm: either way there is no further interest in E and resources are better devoted to other experimental treatments. The triangular test was devised over 50 years ago [13], and has been used extensively in a wide range of studies [14].

Adoption of the GOST design should be subject to approval of a Data and Safety Monitoring Board (DSMB), who consider unblinded data during the ongoing trial. They have the duty to recommend stopping the trial if they feel it unsafe to continue, considering the primary categorisation of status after D days and also data on other endpoints and from patients who have not yet been observed for D days. They will also be asked to confirm any stopping recommendation resulting from the triangular boundaries, taking account of information on patient progress not captured by the primary ordinal response, relevant external information, and indications of major discrepancies in treatment effect across patient subgroups.

The trial will also be overseen by a Steering Committee without access to unblinded trial data. This committee could, however, be provided with data on the sample size and the amount of information V available at each interim analysis. This would provide a reassessment of the relationship between these two quantities, as shown in equation E3 of S1 Text, that does not depend on pre-trial assumptions. To protect the accuracy of the trial, the Steering Committee might authorise a change in the numbers of new patient responses to be collected for each interim analysis to ensure that the increments in V are closer to their intended values. As this would be done without access to unblinded data, no bias would be introduced.

The triangular test itself is very flexible, and the approach can be reworked with different choices for α, power and R, and different numbers and patterns of interim analyses (although the name GOST is reserved for the specific case presented here). Normally distributed data, count data, survival data and other types of response can also be accommodated [1].

## Supporting information

S1 Text Supporting technical details. (DOCX)
