# Correction: GOST: A generic ordinal sequential trial design for a treatment trial in an emerging pandemic

**DOI:** 10.1371/journal.pntd.0006414

**Published:** 2018-04-13

**Authors:** John Whitehead, Peter Horby

The authors of this paper regret that there are errors in the third, fourth, and fifth sentences in the sixth paragraph in the Results section. These errors arise from the computer code presented in [2], for which a corrigendum has been published [3]. No results outside that paragraph are affected. This paragraph should read as follows:

Table 3 presents data from a single simulated run of GOST and Figure 6 shows the resulting plot. This fictitious trial stopped at the 11th interim analysis with 242 patients, and E won. Using the approach described in [2], the one-sided p-value is found to be 0.016. The median unbiased estimate of the log-odds ratio θ is 0.534 with 95% confidence interval (0.048, 1.008). For the odds-ratio R, the median unbiased estimate is 1.71 with 95% confidence interval (1.05, 2.74). The simulation did not generate patient data that would be received by the investigators after this analysis, but in practice results would come in from study patients who were still being followed to 28 days at the time the data for the 11th interim analysis were extracted, and those who were recruited while that analysis was being undertaken. Provided that no change was made to the treatment of these patients, they could be included in a subsequent overrunning analysis [7], and this would become the definitive interpretation of the trial results.
