# Supplementary material for: GOST: A generic ordinal sequential trial design for a treatment trial in an emerging pandemic
Source: PLoS Negl Trop Dis. 2017 Mar 9;11(3):e0005439. doi: 10.1371/journal.pntd.0005439 (PMC5360336; doi:10.1371/journal.pntd.0005439)
Supplement: S1 Text — (DOCX) [file pntd.0005439.s001.docx]

**S1 Text. Supporting technical details**

GOST: A generic ordinal sequential trial design for a treatment trial in an emerging pandemic.

John Whitehead, Peter Horby.

***Definition of the test statistics***

If GOST is applied to a multi-centre trial, then the test statistics used will be stratified by centre. Further factors such as pathogen load and age might be used to create finer strata.

At any interim analysis, data from the g^th^ stratum are as shown in the Table, g = 1, ..., G.

**Table: Counts of patients in each outcome category by treatment for the g^th^ stratum**

| Category | Treatment | | Total |
| --- | --- | --- | --- |
|  | Standard (S) | Experimental (E) |  |
| C_1_ | n_S1g_ | n_E1g_ | n_•1g_ |
| C_2_ | n_S2g_ | n_E2g_ | n_•2g_ |
|  |  |  |  |
| C_k_ | n_Skg_ | n_Ekg_ | n_•kg_ |
| Total | n_S•g_ | n_E•g_ | n_••g_ |

For j = 1, ..., k, let N^Sjg^ = n_Sjg_ + ... + n_Skg_ be the number patients receiving S in stratum g whose outcome is C_j_ or worse, and N_Sjg_ = n_S1g_ + ... + n_Sjg_ the number whose outcome is C_j_ or better, with N^S(k+1)g^ = 0 and N_S0g_ = 0, g = 1, ..., G. Define N^Ejg^ and N_Ejg_ similarly for patients receiving E. A statistic expressing the advantage of E over S is

$Z=\sum_{g=1}^{G} \frac{1}{n_{g}}\sum_{j=1}^{k} n_{\mathrm{Ejg}}\left\{ N^{S\left( j+1 \right)g}-N_{S\left( j-1 \right)g} \right\}$ (E1)

and a statistic representing the amount of information available is

$V=\sum_{g=1}^{G} \frac{n_{Sg}n_{Eg}}{3n_{g}}\left\{ 1-\sum_{j=1}^{k} \left( \frac{n_{\mathrm{jg}}}{ng} \right)^{3} \right\}.$ (E2)

These quantities are stratified versions of equations (2.2) and (2.3) of reference 4 in the main manuscript text. Each is formed by summing separate elements from each stratum to provide within stratum comparisons.

The total number of patients in the trial is n_•••_. To predict the amount of information V accruing from n_•••_ patients in the trial, approximations can be made in (E2). Let τ_g_ denote the proportion of all patients in stratum g, and π_jg_ the proportion (averaged over those on E and S) in stratum g anticipated to lie in C_j_, j = 1, ..., k; g = 1, ..., G. Then, for randomisation in the ratio 1:A within all strata, the predicted value of V, V^(pred)^, is

$V^{\left( \mathrm{pred} \right)}=\mathrm{cn}, where c=\left\{ \frac{A}{3\left( A+1 \right)^{2}}\sum_{g=1}^{G} \tau_{g}\left( 1-\sum_{j=1}^{k} \pi_{\mathrm{jg}}^{3} \right) \right\}.$ (E3)

***Stopping boundaries***

At the i^th^ interim analysis, the statistics Z and V (now denoted by Z_i_ and V_i_ respectively) are computed, and Z_i_ is plotted against V_i_ on the diagram shown in Figure 1 of the main manuscript. The stopping rule is represented by two straight lines. The upper boundary is specified by the equation Z = 6.39903 + 0.21049V and the lower boundary by Z = −6.39903 + 0.63147V. The boundaries meet at V = 30.40. The 20 interim analyses should be conducted following increments in V of 30.40/20 = 1.52.

***The conversion factor, c***

It can be seen from (E3) that the predicted value of V is found from the total sample size n_•••_ by multiplication by a conversion factor c, and that c depends on the proportion of patients in each stratum and their distribution of outcomes. Such factors have been used to obtain the pre-trial forecasts shown in Table 2 of the main manuscript. They depend on both the scenario and the value of R assumed. In the simulations conducted and the illustration provided, it is assumed that there is a single stratum. Before the trial is conducted, investigators assume that Scenario 1 is valid. To make predictions when R = 1, the proportions of outcomes in each of C_1_, C_2_, C_3_ and C_4_ are anticipated to be 0.286, 0.043, 0.214 and 0.457 for both E and S. These values are used in (E3) to yield c = 0.0726. Each observation will increase the amount of information V by 0.0726: conversely 1/c = 13.77 observations will be needed to increase V by 1. To make predictions when R = 2, the outcome distribution for S is assumed to be as above, but that for E is taken to comprise the proportions 0.445, 0.050, 0.209 and 0.296. Averaging over E and S provides the values 0.366, 0.047, 0.211 and 0.376 to be used in (E3). This results in c = 0.074, and 1/c = 13.51. A similar procedure is used in the case R = 1.5. In converting Figure 4 to Figure 5 of the main manuscript, the same procedure is used with R = e^θ^, for every value of θ used in the plot.

As the increase in V between interim analyses is planned to be 1.52, the number of new responses at each interim analysis should be 1.52/c. The simulated trials are planned under Scenario 1, and here the value R = 2 has been adopted for the calculation. Now 1.52/0.074 = 20.5, and rounding to the next larger even integer, the design involves collecting 22 new responses for each interim analysis. The maximum sample size after the full 20 interim analyses will be 440.

***The case of binary data***

The statistics Z and V of equations (E1) and (E2) take particularly simple forms in the case of binary data. Suppose that k = 2, let C_1_ represent success and C_2_ failure, and adopt simpler notation for this special case. At some interim analysis there are responses from n_S_ patients on S, of whom S_S_ have succeeded; and n_E_ patients on E, of whom S_E_ have succeeded. The total number of patients is n = n_S_ + n_E_, the total number of successes is S = S_S_ + S_E_, and the total number of failures is F = n – S. Then, for a single stratum and suppressing the subscript i for interim analysis number,

$Z=\frac{n_{S}n_{E}}{n}\left( \frac{S_{E}}{n_{E}}-\frac{S_{S}}{n_{S}} \right)$ and $V= \frac{n_{S}n_{E}\mathrm{SF}}{n^{3}}$ . (E4)

It can be seen that, in the binary case, Z is simply a multiple of the difference between the success rates on the two treatments and V a simple combination of the information available on each treatment and the split into successes and failures (for example, if no-one has succeeded there is no information on relative success rates). The conversion factor c is given by

$c= \frac{A}{\left( A+1 \right)^{2}}\bar{\pi}\left( 1-\bar{\pi} \right)$, (E5)

where $\bar{\pi}$ denotes the average success probability over E and S. For the binary Scenario 3 with R = 1, c = 0.0583 and 1/c =17.16. The number of new responses at each interim analysis is 1.52/c = 26.08. On this occasion, we rounded down to 26 per interim analysis, with a maximum sample size of 520.
